# Crystal structure of di­chlorido­bis­(*N*,*N*′-di­methyl­thio­urea-κ*S*)mercury(II)

**DOI:** 10.1107/S2056989015015406

**Published:** 2015-08-22

**Authors:** Muhammad Ashraf Shaheen, Aisha Munawar, Haseeba Sadaf, Muhammad Nawaz Tahir, Anvarhusein A. Isab, Saeed Ahmad

**Affiliations:** a Department of Chemistry, University of Sargodha, Sargodha, Punjab, Pakistan; b Department of Chemistry, University of Engineering and Technology, Lahore 54890, Pakistan; c Department of Physics, University of Sargodha, Sargodha, Punjab, Pakistan; d Department of Chemistry, King Fahd University of Petroleum and Minerals, Dhahran 31261, Saudi Arabia

**Keywords:** crystal structure, mercury, di­methyl­thio­urea, hydrogen bonding

## Abstract

The mol­ecular structure of the title compound, [HgCl_2_(C_3_H_8_N_2_S)_2_], has point group symmetry 2, with the twofold rotation axis passing through the Hg^II^ atom. The latter is coordinated by two Cl atoms and two *N*,*N*′-di­methyl­thio­urea (Dmtu) ligands through their S atoms, defining a distorted tetra­hedral coordination sphere with bond angles in the range 102.47 (4)–118.32 (4)°. Intra- and inter­molecular hydrogen bonds of the type N—H⋯Cl with *S*(6) and *R*_2_^2^(12) ring motifs are present. The inter­molecular contacts make up polymeric chains extending parallel to [101].

## Abstract 2

The title compound, [Hg(C_3_H_8_N_2_S)_2_Cl_2_], is isotypic with its Zn and Cd analogues, having the transition metal in a distorted tetra­hedral Cl_2_S_2_ coordination environment.

## Chemical context   

X-ray structural studies of mercury(II) complexes with thio­urea ligands (*L*) or derivatives thereof have shown that in combination with a halide or pseudohalide *X*, some of the complexes exist as mononuclear species [Hg*X*_2_ *L*_2_] (Popović *et al.*, 2000 ▸), while the others exist in a dimeric or polymeric form as [Hg*X*_2_ *L*]_*n*_ (Bell *et al.*, 2001 ▸) in the solid state. In both types of complexes, monomeric (1:2) or polymeric (1:1), the coordination environment around Hg^II^ is distorted tetra­hedral or pseudo-tetra­hedral. We have recently reported the crystal structures of HgCl_2_ and Hg(CN)_2_ complexes with methyl­thio­urea as an auxiliary ligand (Isab *et al.*, 2011 ▸), *N*,*N*′-di­methyl­thio­urea (Malik *et al.*, 2010*a* ▸), *N*,*N*′-di­ethyl­thio­urea (Mufakkar *et al.*, 2010 ▸), *N*,*N*′-di­butyl­thio­urea (Ahmad *et al.*, 2009 ▸) and tetra­methyl­thio­urea (Nawaz *et al.*, 2010 ▸).
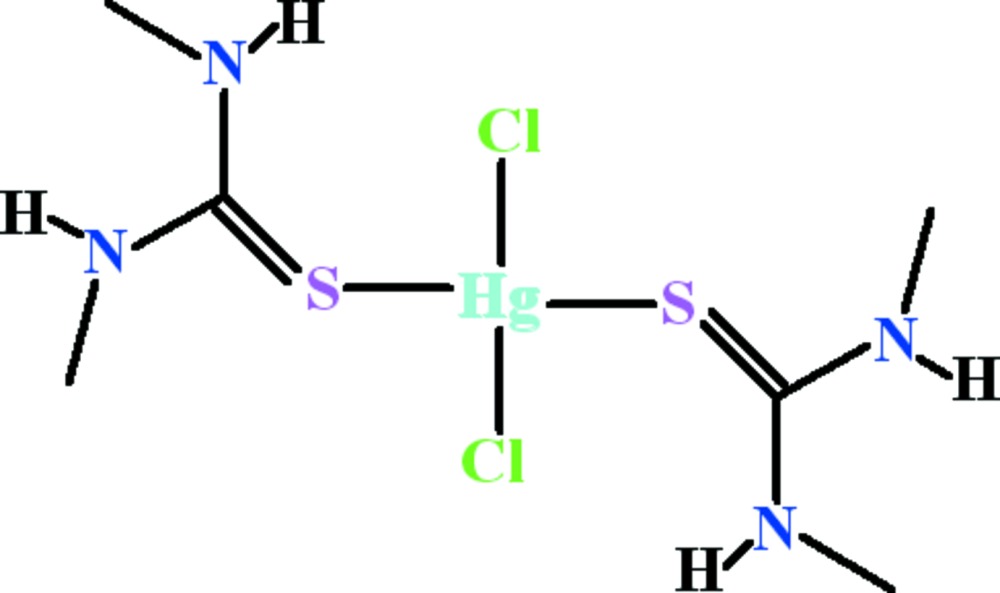


In this article, we report on synthesis and crystal structure of HgCl_2_ with di­methyl­thio­urea (Dmtu) as an additional ligand, [HgCl_2_(C_3_H_8_N_2_S)_2_], (I).

## Structural comments   

**Figure 1:**
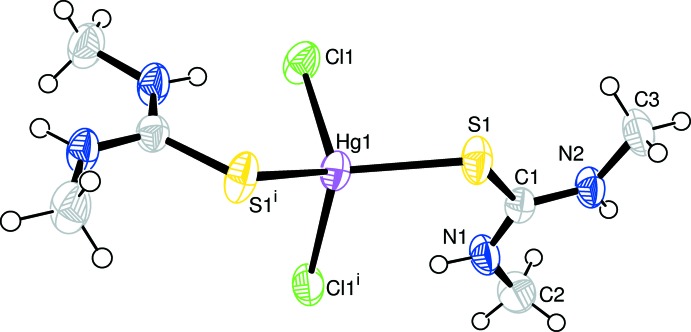
The mol­ecular structure of compound (I). Displacement ellipsoids are drawn at the 50% probability level. H atoms are shown as small circles of arbitrary radius. [Symmetry code: (i) −*x* + 1, *y*, −*z* + 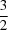.]

The mercury atom in complex (I) lies on a twofold rotation axis (Fig. 1 ▸). It exhibits a distorted tetra­hedral coordination environment defined by two S atoms of symmetry-related Dmtu ligands and two Cl atoms. The S—Hg—S bond angle is 118.32 (4)°. At 102.47 (4)°, the Cl—Hg—Cl bond angle is significantly smaller, which can be attributed to the bulkier Dmtu ligands. The Hg—S, Hg—Cl and other bond lengths (Table 1 ▸) have similar values compared with other [HgCl_2_ *L*_2_] complexes (Ahmad *et al.*, 2009 ▸; Isab *et al.*, 2011 ▸; Malik *et al.*, 2010*a* ▸; Mufakkar *et al.*, 2010 ▸; Popović *et al.*, 2000 ▸). In (I), the N—(C=S)—N skeleton of the Dmtu ligand is essentially planar with an r.m.s. deviation of 0.0135 Å.

## Supra­molecular features   

**Figure 2:**
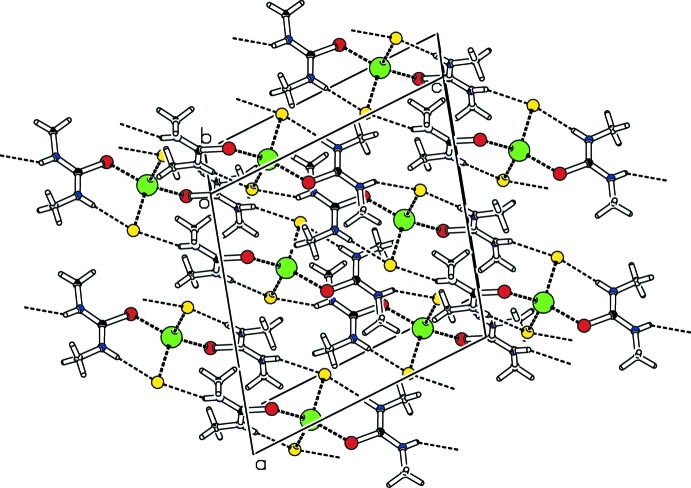
Crystal packing of compound (I) viewed approximately along [010]. N—H⋯Cl hydrogen bonds are shown as dashed lines (see Table 2 ▸ for details).

From a supra­molecular point of view, adjacent mol­ecules are connected by inter­molecular N2—H2⋯Cl1 hydrogen bonds (Table 2 ▸, Fig. 2 ▸) into 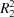
(12) ring motifs (Bernstein *et al.*, 1995 ▸). The supra­molecular chains formed this way extend parallel to [101]. Additional intra­molecular hydrogen bonds N1—H1⋯Cl1 (Table 2 ▸) with *S*(6) loop motifs (Bernstein *et al.*, 1995 ▸) are also present.

## Database survey   

A systematic search in the Cambridge Structural Database (Groom & Allen, 2014 ▸) revealed a total of 25 hits for mercury chloride complexes with thio­urea ligands. The title compound is isotypic with the Zn and Cd analogues [ZnCl_2_(Dmtu)_2_] (Burrows *et al.*, 2004 ▸) and [CdCl_2_(Dmtu)_2_] (Malik *et al.*, 2010*b* ▸) and with [CdBr_2_(Dmtu)_2_] (Ahmad *et al.*, 2011 ▸). The Hg^II^ atom in the structure of (I) shows an equivalent degree of distortion from the tetra­hedral configuration as the metals in [Zn(Dmtu)_2_Cl_2_] and [Hg(tetra­methyl­thio­urea)_2_Cl_2_] (Nawaz *et al.*, 2010 ▸) in which the bond angles at the metal atom vary from 104.35 (2) to 113.30 (2)° and from 104.08 (4) to 120.75 (4)°, respectively. However, in [CdCl_2_(Dmtu)_2_] and [CdBr_2_(Dmtu)_2_], the coordination spheres around Cd deviate only slightly from ideal tetra­hedral values. On the other hand in [Hg(Dmtu)_2_(CN)_2_], the Hg^II^ atom exhibits a severely distorted tetra­hedral coordination sphere with bond angles in the range 94.31 (2) to 148.83 (13)° (Malik *et al.*, 2010*a* ▸).

## Synthesis and crystallization   

For the preparation of title complex, 0.27 g (1 mmol) HgCl_2_ dissolved in 4 ml di­methyl­sulfoxide were mixed with two equivalents of *N*,*N*′-di­methyl­thio­urea in 10 ml aceto­nitrile. After stirring for 15 minutes, the resulting solution was filtered and the filtrate kept at room temperature. After one day colourless crystals were obtained. Yield *ca*. 60%.

## Refinement   

Crystal data, data collection and structure refinement details are summarized in Table 3 ▸. All H atoms were positioned geometrically (C—H = 0.96 Å, N—H= 0.86 Å) and refined as riding with *U*_iso_(H) = 1.5*U*_eq_(C) and *U*_iso_(H) = 1.2*U*_eq_(N).

## Supplementary Material

Crystal structure: contains datablock(s) global, I. DOI: 10.1107/S2056989015015406/wm5202sup1.cif

Structure factors: contains datablock(s) I. DOI: 10.1107/S2056989015015406/wm5202Isup2.hkl

CCDC reference: 1419298

Additional supporting information:  crystallographic information; 3D view; checkCIF report

## Figures and Tables

**Table 1:** Selected bond lengths ()

Hg1S1	2.4622(7)	Hg1Cl1	2.5589(7)

**Table 2:** Hydrogen-bond geometry (, )

*D*H*A*	*D*H	H*A*	*D* *A*	*D*H*A*
N1H1Cl1	0.86	2.37	3.223(3)	170
N2H2Cl1^i^	0.86	2.49	3.270(3)	151

Symmetry code: (i) 
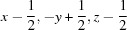
.

**Table 3:** Experimental details

Crystal data	
Chemical formula	[HgCl_2_(C_3_H_8_NS)_2_]
*M*_r_	479.84
Crystal system, space group	Monoclinic, *C*2/*c*
Temperature (K)	296
*a*, *b*, *c* ()	13.1434(12), 8.9971(3), 12.6596(9)
()	107.955(4)
*V* (^3^)	1424.12(17)
*Z*	4
Radiation type	Mo *K*
(mm^1^)	11.45
Crystal size (mm)	0.36 0.18 0.16

Data collection	
Diffractometer	Bruker Kappa APEXII CCD
Absorption correction	Multi-scan (*SADABS*; Bruker, 2007 ▸)
*T*_min_, *T*_max_	0.106, 0.265
No. of measured, independent and observed [*I* > 2(*I*)] reflections	12262, 1721, 1556
*R*_int_	0.031
(sin /)_max_ (^1^)	0.661

Refinement	
*R*[*F* ^2^ > 2(*F* ^2^)], *wR*(*F* ^2^), *S*	0.018, 0.038, 1.06
No. of reflections	1721
No. of parameters	71
H-atom treatment	H-atom parameters constrained
_max_, _min_ (e ^3^)	0.42, 0.32

Computer programs: *APEX2* and *SAINT* (Bruker, 2007 ▸), *SHELXS97* (Sheldrick, 2008 ▸), *SHELXL2014*/6 (Sheldrick, 2015 ▸), *ORTEP-3 for Windows* and *WinGX* (Farrugia, 2012 ▸) and *PLATON* (Spek, 2009 ▸).

## supplementary crystallographic information

### Crystal data

**Table:**

[HgCl_2_(C_3_H_8_NS)_2_]	*F*(000) = 904
*M**_r_* = 479.84	*D*_x_ = 2.238 Mg m^−^^3^
Monoclinic, *C*2/*c*	Mo *K*α radiation, λ = 0.71073 Å
*a* = 13.1434 (12) Å	Cell parameters from 1556 reflections
*b* = 8.9971 (3) Å	θ = 3.0–28.0°
*c* = 12.6596 (9) Å	µ = 11.45 mm^−^^1^
β = 107.955 (4)°	*T* = 296 K
*V* = 1424.12 (17) Å^3^	Lath, colourless
*Z* = 4	0.36 × 0.18 × 0.16 mm

### Data collection

**Table:**

Bruker Kappa APEXII CCD diffractometer	1721 independent reflections
Radiation source: fine-focus sealed tube	1556 reflections with *I* > 2σ(*I*)
Graphite monochromator	*R*_int_ = 0.031
Detector resolution: 7.50 pixels mm^-1^	θ_max_ = 28.0°, θ_min_ = 3.0°
ω scans	*h* = −16→17
Absorption correction: multi-scan (*SADABS*; Bruker, 2007)	*k* = −11→11
*T*_min_ = 0.106, *T*_max_ = 0.265	*l* = −16→16
12262 measured reflections	

### Refinement

**Table:**

Refinement on *F*^2^	Primary atom site location: structure-invariant direct methods
Least-squares matrix: full	Secondary atom site location: difference Fourier map
*R*[*F*^2^ > 2σ(*F*^2^)] = 0.018	Hydrogen site location: inferred from neighbouring sites
*wR*(*F*^2^) = 0.038	H-atom parameters constrained
*S* = 1.06	*w* = 1/[σ^2^(*F*_o_^2^) + (0.0168*P*)^2^ + 0.595*P*] where *P* = (*F*_o_^2^ + 2*F*_c_^2^)/3
1721 reflections	(Δ/σ)_max_ = 0.001
71 parameters	Δρ_max_ = 0.42 e Å^−^^3^
0 restraints	Δρ_min_ = −0.31 e Å^−^^3^

### Special details

**Table:**

Geometry. All esds (except the esd in the dihedral angle between two l.s. planes) are estimated using the full covariance matrix. The cell esds are taken into account individually in the estimation of esds in distances, angles and torsion angles; correlations between esds in cell parameters are only used when they are defined by crystal symmetry. An approximate (isotropic) treatment of cell esds is used for estimating esds involving l.s. planes.
Refinement. Refinement of *F*^2^ against ALL reflections. The weighted *R*-factor *wR* and goodness of fit *S* are based on *F*^2^, conventional *R*-factors *R* are based on *F*, with *F* set to zero for negative *F*^2^. The threshold expression of *F*^2^ > σ(*F*^2^) is used only for calculating *R*-factors(gt) *etc*. and is not relevant to the choice of reflections for refinement. *R*-factors based on *F*^2^ are statistically about twice as large as those based on *F*, and *R*- factors based on ALL data will be even larger.

### Fractional atomic coordinates and isotropic or equivalent isotropic displacement parameters (Å^2^)

**Table:**

	*x*	*y*	*z*	*U*_iso_*/*U*_eq_	
Hg1	0.5000	0.37748 (2)	0.7500	0.04175 (6)	
Cl1	0.60610 (6)	0.19941 (10)	0.66623 (6)	0.05387 (19)	
S1	0.36846 (7)	0.51777 (8)	0.60297 (6)	0.04873 (19)	
N1	0.40138 (19)	0.3089 (3)	0.4670 (2)	0.0464 (6)	
H1	0.4588	0.2910	0.5206	0.056*	
N2	0.24749 (19)	0.4417 (3)	0.4042 (2)	0.0433 (5)	
H2	0.2332	0.3894	0.3446	0.052*	
C1	0.3374 (2)	0.4131 (3)	0.4824 (2)	0.0362 (6)	
C2	0.3824 (3)	0.2226 (4)	0.3672 (3)	0.0553 (8)	
H2A	0.4381	0.1500	0.3772	0.083*	
H2B	0.3819	0.2870	0.3066	0.083*	
H2C	0.3146	0.1732	0.3513	0.083*	
C3	0.1704 (3)	0.5533 (4)	0.4096 (3)	0.0580 (9)	
H3A	0.1572	0.5471	0.4800	0.087*	
H3B	0.1048	0.5371	0.3509	0.087*	
H3C	0.1978	0.6501	0.4015	0.087*	

### Atomic displacement parameters (Å^2^)

**Table:**

	*U*^11^	*U*^22^	*U*^33^	*U*^12^	*U*^13^	*U*^23^
Hg1	0.04156 (10)	0.04908 (9)	0.02828 (9)	0.000	0.00146 (7)	0.000
Cl1	0.0503 (4)	0.0684 (5)	0.0368 (4)	0.0220 (4)	0.0044 (3)	−0.0017 (3)
S1	0.0591 (5)	0.0440 (4)	0.0315 (4)	0.0151 (3)	−0.0031 (3)	−0.0054 (3)
N1	0.0395 (14)	0.0570 (14)	0.0344 (13)	0.0126 (11)	−0.0007 (11)	−0.0056 (11)
N2	0.0425 (14)	0.0480 (12)	0.0308 (12)	0.0096 (11)	−0.0011 (11)	−0.0027 (11)
C1	0.0366 (16)	0.0375 (13)	0.0314 (14)	0.0001 (10)	0.0059 (12)	0.0026 (10)
C2	0.056 (2)	0.0626 (19)	0.0429 (18)	0.0096 (15)	0.0091 (16)	−0.0154 (15)
C3	0.051 (2)	0.067 (2)	0.0443 (19)	0.0258 (17)	−0.0017 (15)	−0.0002 (16)

### Geometric parameters (Å, º)

**Table:**

Hg1—S1	2.4622 (7)	N2—C3	1.444 (4)
Hg1—S1^i^	2.4622 (7)	N2—H2	0.8600
Hg1—Cl1	2.5589 (7)	C2—H2A	0.9600
Hg1—Cl1^i^	2.5589 (7)	C2—H2B	0.9600
S1—C1	1.732 (3)	C2—H2C	0.9600
N1—C1	1.313 (4)	C3—H3A	0.9600
N1—C2	1.438 (4)	C3—H3B	0.9600
N1—H1	0.8600	C3—H3C	0.9600
N2—C1	1.312 (4)		
			
S1—Hg1—S1^i^	118.32 (4)	N2—C1—S1	118.1 (2)
S1—Hg1—Cl1	110.67 (2)	N1—C1—S1	122.1 (2)
S1^i^—Hg1—Cl1	106.79 (3)	N1—C2—H2A	109.5
S1—Hg1—Cl1^i^	106.79 (3)	N1—C2—H2B	109.5
S1^i^—Hg1—Cl1^i^	110.67 (2)	H2A—C2—H2B	109.5
Cl1—Hg1—Cl1^i^	102.47 (4)	N1—C2—H2C	109.5
C1—S1—Hg1	107.88 (9)	H2A—C2—H2C	109.5
C1—N1—C2	124.8 (3)	H2B—C2—H2C	109.5
C1—N1—H1	117.6	N2—C3—H3A	109.5
C2—N1—H1	117.6	N2—C3—H3B	109.5
C1—N2—C3	125.7 (3)	H3A—C3—H3B	109.5
C1—N2—H2	117.2	N2—C3—H3C	109.5
C3—N2—H2	117.2	H3A—C3—H3C	109.5
N2—C1—N1	119.8 (3)	H3B—C3—H3C	109.5
			
C3—N2—C1—N1	−179.5 (3)	C2—N1—C1—S1	−177.2 (2)
C3—N2—C1—S1	−0.5 (4)	Hg1—S1—C1—N2	159.3 (2)
C2—N1—C1—N2	1.8 (5)	Hg1—S1—C1—N1	−21.6 (3)

Symmetry code: (i) −*x*+1, *y*, −*z*+3/2.

### Hydrogen-bond geometry (Å, º)

**Table:**

*D*—H···*A*	*D*—H	H···*A*	*D*···*A*	*D*—H···*A*
N1—H1···Cl1	0.86	2.37	3.223 (3)	170
N2—H2···Cl1^ii^	0.86	2.49	3.270 (3)	151

Symmetry code: (ii) *x*−1/2, −*y*+1/2, *z*−1/2.

## References

[bb1] Ahmad, S., Altaf, M., Stoeckli-Evans, H., Isab, A. A., Malik, M. R., Ali, S. & Shuja, S. (2011). *J. Chem. Crystallogr.* **41**, 1099–1104.

[bb2] Ahmad, S., Sadaf, H., Akkurt, M., Sharif, S. & Khan, I. U. (2009). *Acta Cryst.* E**65**, m1191–m1192. 10.1107/S1600536809035594 PMC2970451 21577723

[bb3] Bell, N. A., Branston, T. N., Clegg, W., Parker, L., Raper, E. S., Sammon, C. & Constable, C. P. (2001). *Inorg. Chim. Acta*, **319**, 130–136.

[bb4] Bernstein, J., Davis, R. E., Shimoni, L. & Chang, N.-L. (1995). *Angew. Chem. Int. Ed. Engl.* **34**, 1555–1573.

[bb5] Bruker (2007). *APEX2*, *SAINT* and *SADABS*. Bruker AXS Inc., Madison, Wisconsin, USA.

[bb6] Burrows, A. D., Harrington, R. W. & Mahon, M. F. (2004). *Acta Cryst.* E**60**, m1317–m1318.

[bb7] Farrugia, L. J. (2012). *J. Appl. Cryst.* **45**, 849–854.

[bb8] Groom, C. R. & Allen, F. H. (2014). *Angew. Chem. Int. Ed.* **53**, 662–671. 10.1002/anie.201306438 24382699

[bb9] Isab, A. A., Fettouhi, M., Malik, M. R., Ali, S., Fazal, A. & Ahmad, S. (2011). *Russ. J. Coord. Chem.* **37**, 180–185.

[bb14] Malik, M. R, Ali, S., Ahmad, S., Altaf, M. & Stoeckli-Evans, H. (2010*b*). *Acta Cryst.* E**66**, m1060–m1061. 10.1107/S1600536810030424 PMC3008114 21588485

[bb10] Malik, M. R., Ali, S., Fettouhi, M., Isab, A. A. & Ahmad, S. (2010*a*). *J. Struct. Chem.* **51**, 976–979.

[bb11] Mufakkar, M., Tahir, M. N., Sadaf, H., Ahmad, S. & Waheed, A. (2010). *Acta Cryst.* E**66**, m1001–m1002. 10.1107/S1600536810028825 PMC3007422 21588084

[bb12] Nawaz, S., Sadaf, H., Fettouhi, M., Fazal, A. & Ahmad, S. (2010). *Acta Cryst.* E**66**, m952. 10.1107/S1600536810028138 PMC3007291 21588182

[bb13] Popović, Z., Pavlović, G., Matković-Čalogović, D., Soldin, Ž., Željka, , Rajić, M., Vikić-Topić, D. & Kovaček, D. (2000). *Inorg. Chim. Acta*, **306**, 142–152.

[bb15] Sheldrick, G. M. (2008). *Acta Cryst.* A**64**, 112–122. 10.1107/S0108767307043930 18156677

[bb16] Sheldrick, G. M. (2015). *Acta Cryst.* C**71**, 3–8.

[bb17] Spek, A. L. (2009). *Acta Cryst.* D**65**, 148–155. 10.1107/S090744490804362X PMC2631630 19171970

